# Supplementary material for: Mitochondrial dysfunction drives a neuronal exhaustion phenotype in methylmalonic aciduria
Source: Commun Biol. 2025 Mar 11;8:410. doi: 10.1038/s42003-025-07828-z (PMC11897345; doi:10.1038/s42003-025-07828-z)
Supplement: Supplementary file 2 — Description of Supplement Data Files [file 42003_2025_7828_MOESM2_ESM.pdf]

Description of Additional Supplementary Files

File name: Supplementary Data

Description: The source data behind each graph in the paper separated by tabs.
