# Supplementary material for: Mitochondrial dysfunction drives a neuronal exhaustion phenotype in methylmalonic aciduria
Source: Commun Biol. 2025 Mar 11;8:410. doi: 10.1038/s42003-025-07828-z (PMC11897345; doi:10.1038/s42003-025-07828-z)
Supplement: Supplementary file 4 — Reporting Summary [file 42003_2025_7828_MOESM4_ESM.pdf]

nature portfolio

Corresponding author(s): *Double-anonymous peer review submissions: write DAPR and your manuscript number here instead of author names.*

Last updated by author(s): *YYYY-MM-DD*

Reporting Summary

Nature Portfolio wishes to improve the reproducibility of the work that we publish. This form provides structure for consistency and transparency in reporting. For further information on Nature Portfolio policies, see our Editorial Policies and the Editorial Policy Checklist.

Statistics

For all statistical analyses, confirm that the following items are present in the figure legend, table legend, main text, or Methods section.

n/a	Confirmed	
☐	☒	The exact sample size (*n*) for each experimental group/condition, given as a discrete number and unit of measurement
☐	☒	A statement on whether measurements were taken from distinct samples or whether the same sample was measured repeatedly
☐	☒	The statistical test(s) used AND whether they are one- or two-sided<br>*Only common tests should be described solely by name; describe more complex techniques in the Methods section.*
☐	☒	A description of all covariates tested
☐	☒	A description of any assumptions or corrections, such as tests of normality and adjustment for multiple comparisons
☐	☒	A full description of the statistical parameters including central tendency (e.g. means) or other basic estimates (e.g. regression coefficient) AND variation (e.g. standard deviation) or associated estimates of uncertainty (e.g. confidence intervals)
☐	☒	For null hypothesis testing, the test statistic (e.g. *F*, *t*, *r*) with confidence intervals, effect sizes, degrees of freedom and *P* value noted<br>*Give P values as exact values whenever suitable.*
☒	☐	For Bayesian analysis, information on the choice of priors and Markov chain Monte Carlo settings
☒	☐	For hierarchical and complex designs, identification of the appropriate level for tests and full reporting of outcomes
☐	☒	Estimates of effect sizes (e.g. Cohen's *d*, Pearson's *r*), indicating how they were calculated

Our web collection on statistics for biologists contains articles on many of the points above.

Software and code

Policy information about availability of computer code

Data collection	Leica Application Suite version, 2.8.1; HEKA Patchmaster; MacCoss Lab Skyline version, 20.2; LightCycler 480 1.5.0 SP4, QuantStudio 7; CLSM Leica SP5 resonant APD
Data analysis	Infinium Global Screening Array v3.0; IgorPro software (WaveMetrics, version 6.37); miniML in Python (version 3.9); R Studio (version 2022.12.0+353); fastp v0.02; Kallisto v0.46.1; edgeR v3.42; CybersortX; CIBERSORTx; PRISM GraphPad (v.5.03); Fiji, ImageJ 2.15;

For manuscripts utilizing custom algorithms or software that are central to the research but not yet described in published literature, software must be made available to editors and reviewers. We strongly encourage code deposition in a community repository (e.g. GitHub). See the Nature Portfolio guidelines for submitting code & software for further information.

Data

Policy information about availability of data

All manuscripts must include a data availability statement. This statement should provide the following information, where applicable:

- Accession codes, unique identifiers, or web links for publicly available datasets
- A description of any restrictions on data availability
- For clinical datasets or third party data, please ensure that the statement adheres to our policy

Contributing data to the manuscript has been uploaded as "Supplementary Data". Where applicable scripts and codes are provided with links to GitHub. Bulk RNAseq data has been uploaded to GEO and the accession code is provided in the manuscript.

Research involving human participants, their data, or biological material

Policy information about studies with human participants or human data. See also policy information about sex, gender (identity/presentation), and sexual orientation and race, ethnicity and racism.

Reporting on sex and gender	Sex of cell lines is reported.
Reporting on race, ethnicity, or other socially relevant groupings	N/A
Population characteristics	N/A
Recruitment	Participants provided tissue for diagnostic and research purposes, participants were selected on the grounds of being affected by methylmalonic aciduria.
Ethics oversight	Cantonal Ethics Commission of Zurich (KEK-ZH-Nr. 2014-0211, amendment PB_2020-00053).

Note that full information on the approval of the study protocol must also be provided in the manuscript.

Field-specific reporting

Please select the one below that is the best fit for your research. If you are not sure, read the appropriate sections before making your selection.

☒ Life sciences ☐ Behavioural & social sciences ☐ Ecological, evolutionary & environmental sciences

For a reference copy of the document with all sections, see nature.com/documents/nr-reporting-summary-flat.pdf

Life sciences study design

All studies must disclose on these points even when the disclosure is negative.

Sample size	Two patients are included; this is a rare disease.
Data exclusions	No data excluded.
Replication	Technical and biological replicates are made clear in the manuscript.
Randomization	No randomization performed. Allocation into groups determined by mutation in MMUT gene.
Blinding	Initial electrophysiology experiements were conducted in a single-blind method.

Reporting for specific materials, systems and methods

We require information from authors about some types of materials, experimental systems and methods used in many studies. Here, indicate whether each material, system or method listed is relevant to your study. If you are not sure if a list item applies to your research, read the appropriate section before selecting a response.

Materials & experimental systems

n/a	Involved in the study	
☐	☒	Antibodies
☐	☒	Eukaryotic cell lines
☒	☐	Palaeontology and archaeology
☒	☐	Animals and other organisms
☒	☐	Clinical data
☒	☐	Dual use research of concern
☒	☐	Plants

Methods

n/a	Involved in the study	
☒	☐	ChIP-seq
☒	☐	Flow cytometry
☒	☐	MRI-based neuroimaging

Antibodies

Antibodies used	Refer to Supplementary Table 4.
Validation	MMUT was validated in-house on MMUT-KO cell lines. These data are present in supplementary data.

Eukaryotic cell lines

Policy information about cell lines and Sex and Gender in Research

Cell line source(s)	Ct1, ATCC:424 CRL-2522; Ct2, NHLBli003-A, RRID: CVCL_1E78; HEK, CRL-1573
Authentication	Authentication of iPSC lines Ct1, Pt1, Pt2 at multiple levels including aGCH and germ-layer differentiation.
Mycoplasma contamination	Cell lines were not tested for mycoplasma.
Commonly misidentified lines (See ICLAC register)	N/A

Plants

Seed stocks	N/A
Novel plant genotypes	N/A
Authentication	N/A
